# Exploring the Adoption of Precision Agriculture for Irrigation in the Context of Agriculture 4.0: The Key Role of Internet of Things

**DOI:** 10.3390/s20247091

**Published:** 2020-12-11

**Authors:** Sergio Monteleone, Edmilson Alves de Moraes, Brenno Tondato de Faria, Plinio Thomaz Aquino Junior, Rodrigo Filev Maia, André Torre Neto, Attilio Toscano

**Affiliations:** 1 School of Business Administration, Centro Universitário FEI, São Paulo 01525-000, Brazil; edmilson@fei.edu.br; 2 School of Electrical Engineering, Centro Universitário FEI, São Bernardo do Campo 09850-901, Brazil; betondato@fei.edu.br (B.T.d.F.); plinio.aquino@fei.edu.br (P.T.A.J.); 3 Centre of Regional and Rural Futures, Deakin University, Hanwood 2680, Australia; r.filevmaia@deakin.edu.au; 4 Brazilian Agricultural Research Corporation (EMBRAPA), São Carlos 13560-970, Brazil; andre.torre@embrapa.br; 5 Department of Agricultural and Food Sciences (DISTAL), University of Bologna, 40127 Bologna, Italy; attilio.toscano@unibo.it

**Keywords:** precision agriculture, adoption, irrigation, agriculture 4.0, Internet of things, sensing technologies, weather station, satellite, farmer behavior, operations management

## Abstract

In recent years, the concept of Agriculture 4.0 has emerged as an evolution of precision agriculture (PA) through the diffusion of the Internet of things (IoT). There is a perception that the PA adoption is occurring at a slower pace than expected. Little research has been carried out about Agriculture 4.0, as well as to farmer behavior and operations management. This work explores what drives the adoption of PA in the Agriculture 4.0 context, focusing on farmer behavior and operations management. As a result of a multimethod approach, the factors explaining the PA adoption in the Agriculture 4.0 context and a model of irrigation operations management are proposed. Six simulation scenarios are performed to study the relationships among the factors involved in irrigation planning. Empirical findings contribute to a better understanding of what Agriculture 4.0 is and to expand the possibilities of IoT in the PA domain. This work also contributes to the discussion on Agriculture 4.0, thanks to multidisciplinary research bringing together the different perspectives of PA, IoT and operations management. Moreover, this research highlights the key role of IoT, considering the farmer’s possible choice to adopt several IoT sensing technologies for data collection.

## 1. Introduction

Agriculture is changing in recent years and, in the same way as the industry, is forced to modernize its work methodologies and to take advantage of opportunities offered by the Internet of things (IoT) [1]. Despite this progress, it still cannot be claimed that precision agriculture (PA) has been widely established [2]. There is a perception that the adoption of PA is occurring at a slower pace than expected [3]. A confirmation of this perception can be found in the abundant literature that addresses the analysis of the factors affecting the adoption of PA, such as the recent studies of [3,4,5,6].

IoT plays a key role in agriculture since the sensors can measure several quantities continuously, and by using the cloud processing power [7], it is possible to create models to evaluate the crop development and soil resources and water availability to support the decision-making process or even agriculture automation. Such models can evaluate evapotranspiration [8] or soil properties and their dynamics or even evaluate water demands in paddy areas. Data from crop obtained by IoT sensors and meshed up with data gathered by additional IoT devices, such as drones and remote sensing devices, make it possible to correlate a multitude of parameters that helps in a broader crop understanding and to better approach the crop growth dynamics. The IoT sensing technologies make room for innovative ways to better use natural resources like water in irrigation [9,10].

IoT is also one of the key technologies for Agriculture 4.0 [11]. This concept appeared at the beginning of the 21st century, as an evolution of the PA concept through the diffusion of IoT [12]. Little research has been carried out about Agriculture 4.0 and, in particular, on operations management in this context [13,14]. Agriculture 4.0 is still restricted and put off in theory and limited to some pioneering companies [15]. This means that a more in-depth analysis is needed to understand what Agriculture 4.0 is [16].

Several studies highlight the potential of Agriculture 4.0, such as improvements in planning and control [17], intelligent use of data collected by using advanced technologies mounted on board tractors, mobile ground robots, unmanned aerial vehicles (UAVs) and satellites [18] and sustainable growth [11]. Others highlight the challenges to be solved. For example, data by its nature in a scenario of Agriculture 4.0 becomes complex to manage both in terms of size and complexity of the analysis to be carried out [18]. A few mention some factors related to operation management, like the mobility of production facilities and coverage of agricultural fields [1,17]. However, these studies do not investigate the relationship between factors and the adoption of PA in the context of Agriculture 4.0. Some researchers have suggested potential areas of investigation for studying the PA in the 4.0 era. [12], to broaden the understanding of determinant factors in the adoption recommends studying farmer behavior, the field which has as its reference to the theory of planned behavior (TPB) [19]. TPB has been applied in agriculture by some researchers, like [20], but not yet in the Agriculture 4.0 context. Ref. [21] suggests carrying out an analysis regarding whether the inspiration for the concept of Industry 4.0 can facilitate the establishment of operational solutions to explain the unexplored potential of PA in agricultural operations.

Therefore, this research seeks to contribute to filling these gaps by answering the following question: What drives the adoption of precision agriculture in the context of Agriculture 4.0, focusing on farmer behavior and operations management? To answer this question, this research aims to achieve the following objectives: exploring the factors that can affect the adoption of precision agriculture in the context of Agriculture 4.0; proposing a model to understand and formalize agricultural operations management based on identified factors; performing simulation scenarios, to study the relationships among the identified factors that allow the design of the model of agricultural operations management. This model and the simulation results support the development of theory on the adoption of PA in the context of Agriculture 4.0.

Considering the general lack of theory on Agriculture 4.0, this research adopts a multimethod approach consisting of interviews with experts, case studies, modeling techniques and simulations. Semi-structured interviews with experts [22] and case studies [23] are carried out to explore the factors that can affect the adoption investigated. Empirical findings are used to ground the model of agricultural operations management and the simulation scenarios [24,25,26], which guide in supporting the development of theory on the adoption of PA in the context of Agriculture 4.0. The simulation allows the elaboration of theories and the accomplishment of exploratory works [27]. Still, in an era where more data are available, it is possible to create simulation models based on real data, using IoT and sensors [28]. The operations investigated are those relating to irrigation, in line with the international project SWAMP (smart water management platform), of which this work is part. This project involves pilots in Brazil, Italy and Spain, focusing on different crop types and irrigation techniques. SWAMP project intends to bring the concept of IoT for precision irrigation [7]. Aligned to disseminate the results of the SWAMP project, this work includes the irrigation of the açaí palm, a plant native from the Amazon region, whose cultivation is expanding in upland areas. Açaí palm fruit has been showing increasing importance in exports to European, Asian and North American countries [29,30].

To model the agricultural operations management related to irrigation, the integration definition for function modeling (IDEF0) methodology is used. Farm enterprises are complex systems that need to be modeled to facilitate knowledge capitalization and information system design. [31]. IDEF0 is used to identify the system components, data requirements, the flow of information and objects among the components [32]. The modeling of operations management, in particular, planning, scheduling and control, through IDEF0, has been applied by several researchers in the industrial sector [33,34]. However, IDEF0 has rarely been used in the agricultural sector. Examples of applications are the studies of [31] and [35].

Since IDEF0 models attempt to capture the functional components of an enterprise or a system, rather than temporal constraints and flow [25], IDEF0 methodology, as a static model, can be used to create simulations for dynamic analysis [36]. Examples of applications combining the IDEF0 methodology and simulation are the works of [37] and of [32]. The simulation was applied to study the technology adoption for agriculture by some researchers, such as [38].

To perform the simulation scenarios, irrigation planning is chosen since it involves the definitions of water requirements and of irrigation time, which are two priority decisions in irrigation water management. These decisions also have a direct effect on the efficiency of water use [39,40]. Another important parameter in the study of irrigation planning is the reference evapotranspiration that can be used with different crop characteristics to produce proper crop water requirements [8,41]. The crop water requirements are necessary for water resources planning and irrigation water management [42,43,44]. There are several methods to estimate the reference evapotranspiration, despite the widespread of the FOAM model due to its consistency in different climatic regions [41,43,45].

This study is ambitious since, in contrast to previous research on Agriculture 4.0, focused mainly on automation, robotics, food and sustainability, explores farmer behavior and operations management. As a result of empirical research, this work extends the current knowledge on Agriculture 4.0, proposing a list of categories and factors that can explain the adoption of PA in the context of Agriculture 4.0. In contrast to previous research on agriculture, the proposed model of irrigation operations management differentiates between irrigation planning and irrigation scheduling, highlighting the role of irrigation resources. The simulation results allow studying the relationships among some parameters involved in irrigation planning, contributing to a better understanding of what Agriculture 4.0 is. This work also contributes to the Agriculture 4.0 discussion. The access to data and irrigation planning can be improved through the possibility the farmer must adopt several IoT sensing technologies. This highlights the key role of IoT in the evolution of PA.

The purpose of this work is significant due to a multidisciplinary approach. By combining the competencies of PA, IoT and operations management, this work allows us to progress in the knowledge of Agriculture 4.0, bringing together different perspectives. These perspectives lead to propose a definition of what Agriculture 4.0 is as a result of empirical findings and based on the definitions of Agriculture 4.0, PA and IoT. Moreover, this work leads to expand the possibilities of the IoT in the domain of PA, also concerning the irrigation operations management.

In the remainder of this paper, Section 2 introduces the related work. Section 3 introduces the multimethod approach, while Section 4 presents the results of this study. Section 5 discusses these findings and presents the limitations and further research. Finally, Section 6 draws conclusions.

## 2. Related Work

Many evaluation criteria shape the adoption of novel information and communication technology in general [46]. In this section, related work is presented to explore the factors that can affect the adoption of PA in the context of Agriculture 4.0.

### 2.1. Precision Agriculture and Factors Affecting the Adoption

In the literature, it is possible to find more than 20 definitions of precision agriculture. This lack of a clear and shared definition makes it difficult to study the adoption [3]. Moreover, the factors affecting the adoption rates depend on the type of PA technologies [5], which are many, such as remote sensing (including soil sensing, satellite remote sensing and proximal remote sensing), yield monitoring and mapping, variable rate technology, grid or zone soil sampling [3,47]. However, the International Society for precision agriculture (ISPA) recently recognized the following as the official definition of PA: “*Precision Agriculture is a management strategy that gathers, processes and analyzes temporal, spatial and individual data and combines it with other information to support management decisions according to estimated variability for improved resource use efficiency, productivity, quality, profitability and sustainability of agricultural production*” [48].

The abundant literature that addresses the study of the factors affecting the PA adoption focuses on the analysis of farmer and farm characteristics, of economic factors and PA technology characteristics [6,49,50]. The factors related to the agricultural operations management are few studied, and the factors related to Agriculture 4.0 are practically absent, as well as the application of the TPB. Most works apply the survey research method, such as [6]. Qualitative research methods were applied by a few researchers to study the PA adoption, as highlighted by [4].

### 2.2. IoT Sensing Technologies in Precision Agriculture

There are several kinds of IoT sensing technologies being used to estimate the use of water in the agriculture and crop parameters. The parameters that these IoT sensing technologies make available for irrigation water management are summarized in Table 1.

The use of soil probes allows obtaining several key soil parameters like soil moisture, soil salinity and soil temperature at several depths [51]. Local weather stations are devices to capture local environmental data as air temperature and air relative humidity, important parameters to evaluate evapotranspiration. Both sources of data make it possible to evaluate the effects of weather and soil in the water requirements because they indirectly capture the dynamic crop water demands. Other relevant parameters as canopy temperature, vegetation indexes such as normalized difference vegetation index (NDVI) and growing degree days (GDD) [59] can be obtained from infrared and near-infrared bands (B4, B6, B8 bands) of the electromagnetic spectrum [60,61]. Those combined parameters may produce a crop model with a minimum of estimated parameters [51]. All those data may be combined through GPS coordinates and time parameters to estimate the amount of water being used by the crop and estimate water requirements.

The parameters shown in Table 1 are considered in the empirical investigation, in the exploration of factors that can affect the PA adoption in the Agriculture 4.0 context.

### 2.3. Agriculture 4.0 and Factors Related to Operations Management

At present, Agriculture 4.0 is a vague and poorly defined term. Some authors use in the same way Agriculture 4.0 and smart farming or Farming 4.0 or digital agriculture [2,12,15], some authors use Agriculture 4.0 to refer to the fourth agricultural revolution [62], other authors use Agriculture 4.0 as an evolution of Industry 4.0 [11,15,17] or as an evolution of the precision agriculture [2,11,15].

Some recent works have contributed to a better understanding of the concepts of digitalization in agriculture, smart farming and Agriculture 4.0. Digitalization in agriculture implies that management tasks on-farm and off-farm focus on different sorts of data, using sensors, machines, drones and satellites to make more timely or accurate decisions. The concepts of smart farming, precision agriculture, digital agriculture, Agriculture 4.0 are emerged to express different forms of digitalization in the agricultural sector [63]. Smart farming represents the use of smart, data-rich ICT-services and applications, in combination with advanced hardware (in tractors, greenhouses, etc.). Smart farming extends the precision agriculture concept since the existing tasks for management and decision-making based on data are enhanced by context, situation and location awareness [64]. Smart farming includes three main categories of technologies: farm management information systems (FMIS), precision agriculture (PA) and agricultural automation and robotics [65].

As for Agriculture 4.0, recently [16] propose a holistic definition, based on a systematic literature review, taking a multiperspective approach and covering the entire agricultural and food value chain. In line with the scope of the SWAMP project, focused on bringing the IoT concept for precision irrigation in farm pilots, in this work, the definition of [16] is used, but limited to the boundaries of the single farm: “*Agriculture 4.0 is the evolution of precision agriculture, realized through the automated collection, integration and analysis of previously separated data silos coming from the field, equipment sensors and other third-party sources, enabled by the use of smart and digital technologies of Industry 4.0, making in this way possible the generation of knowledge, to support the farmer in the decision-making process in the farm enterprise*”.

Research on Agriculture 4.0 is mainly concentrated on automation and robotics [66,67], food and sustainability [11,14,62]. While studies on operations management, inspired by the concept of Industry 4.0, are limited. Operations management in agriculture deals with the design, planning, scheduling and execution of machine and human operations. An operation in agriculture is generally seen as the link between resources, materials processed, and material produced. Agricultural operations may, for instance, be tillage, seeding, fertilizing, plant care, irrigation and harvesting [21,68].

The inspiration for Industry 4.0 identifies some advantages that can be introduced in agriculture, such as improvements in agricultural planning and control [17]. But, the wave of Industry 4.0 also provides new challenges that traditional farming has to overcome [69]. When compared to classic industrial production, agricultural field operations interact with a biologically active system. Industrial production takes place in close, well-defined environments in which performance data can be measured by deterministic matters. In agricultural operations, by the uncertain nature, many adjustments are possible to optimize the operational methods [21]. The production process in agriculture is different from the industrial one in several aspects: preponderant role of the environment and inherent uncertainty and risk (e.g., crop growth, weather conditions); large time constants of planning procedure; complexity in evaluating risky decisions [68]. The high degree of mobility of the production facilities makes planning and control more difficult than in industrial environments. Even the availability and bandwidth of wireless connections are subject to disturbing influences, making constant communication difficult [17]. Moreover, wireless communication technology must provide coverages ranging from tens of meters to several kilometers since sensors could be deployed far and in remote locations within the crop field [1].

The advantages and challenges analyzed in this section highlight some factors related to agricultural operations management that are considered in the empirical investigation.

### 2.4. Farmer Behavior and Factors Affecting the Adoption

To explore the farmer behavior, the theory of planned behavior (TPB) is applied, which has been widely applied to study behavior in different domains, such as innovative technologies for mobility [70], technology adoption in agriculture [71], water-saving measures for irrigation [72]. Behavioral intention to use products or technologies can be used as an indicator of their adoption [46].

The TPB is a theory developed to predict and explain behaviors that are not under complete volitional control. Intentions are assumed to capture the motivational factors that influence behavior. Intentions to perform behaviors can be predicted with an accuracy of attitude (the degree to which a person has a favorable or unfavorable evaluation of the behavior), subjective norm (perceived social pressure to perform or not perform the behavior) and perceived behavioral control (perceived ease or difficulty of carrying out the behavior, presence or absence of requisite resources and opportunities). The more favorable the attitude and the subjective norm and the larger the perceived behavioral control, the stronger is the intention to perform the behavior under consideration [19]. For performing a given behavior, the person believes that the advantages outweigh the disadvantages [73]. Moreover, the more resources and opportunities individuals believe they possess and the fewer obstacles or impediments they anticipate, the greater should be their perceived control over the behavior [19]. In addition to the three predictors, domain-specific factors are both useful and important for gaining a complete understanding of any behavior [73].

In rural studies, attitude is a central factor concerning the understanding of farmers’ decision-making processes [74]. Regarding the adoption process, innovations could be successfully established if farmers see clear benefits on them [75]. Other factors specific to the agricultural domain are demographic characteristics, farm size, conservativeness, access to data and access to the market [20,21,72,76,77,78]. Studies analyzing the factors related to operations are rare, such as the work of [79].

Since the purpose of this work is to study the adoption in farms, subjective norm is considered outside the scope.

## 3. Multimethod Approach

**Figure 1:**
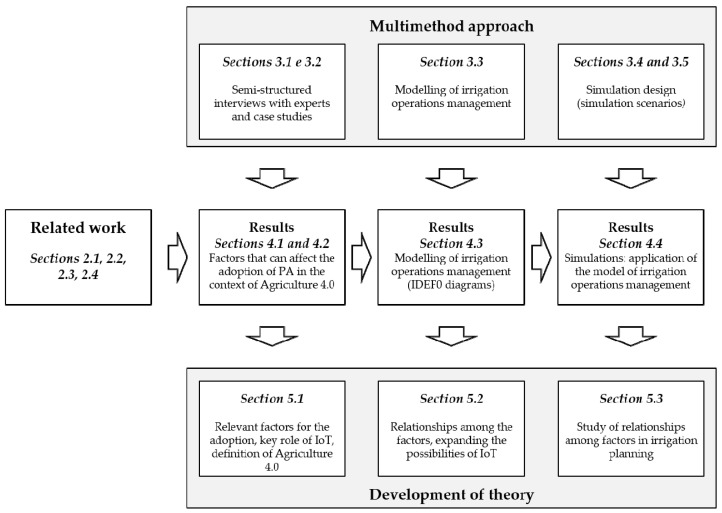
Flowchart of the multimethod approach.

This section presents the multimethod approach adopted in this study, consisting of interviews with experts, case studies, modeling techniques and simulation. The flowchart of the multimethod approach is shown in Figure 1, which enumerates every step taken in this approach.

Considering the general lack of theory on Agriculture 4.0, this research adopts an exploratory multimethod approach. Semi-structured interviews with experts [22] and case studies [23] are carried out to ground the model of agricultural operations management and the simulation scenarios [24,25,26,32]. The purpose of this approach is to produce reliable insights from the empirical investigation, through exploratory interviews and case studies, integrated with relevant literature. Empirical research can provide a strong foundation for making realistic assumptions in mathematical and simulation modeling research in operations management [80], to explore and better understand the emerging and contemporary phenomena of Agriculture 4.0 in its natural settings and to develop theory [23,81].

### 3.1. Semi-Structured Interviews with Experts

The expert interviews are used to complement the case studies beforehand for modeling agricultural operations management and for orientation in the new field of Agriculture 4.0 [22]. The expert interviews are also used to support simulation modeling from empirical findings [82]. Due to the interdisciplinary nature of this research [83], experts were selected for their knowledge of agribusiness, farm management, irrigation, precision agriculture and operations management. The selection of ten interviewees in Brazil was based on purposive sampling [22]. For this, three information-rich experts were selected, whose interviews illuminated the research question. The snowball sampling technique was adopted to select other experts [84]. Six interviews were conducted face-to-face, and four were conducted remotely. Table 2 shows the expert profiles. To protect their identities, they will be referred to as expert A, B, C, D, E, F, G, H, I and L.

The questionnaire used in semi-structured interviews was based on the theory of planned behavior [19,73,85], focused on irrigation and agriculture [38], operations management [32,33,34], including questions about the challenges related to inspiration for Industry 4.0 [1,17,21,68]. The questionnaire is shown in Table A1 in Appendix A. The questionnaire consists of two parts: open questions for each TPB predictor in scope (attitude, perceived behavioral control) and additional questions focused on operations management in irrigation. Considering the exploratory nature of the research, to reduce response bias, the definition of Agriculture 4.0 used in this work was not provided to interviewees [22,86]. A pilot interview was conducted for testing the questionnaire [80]. All the interviews were taped and transcribed. Moreover, field notes were taken.

### 3.2. Case Studies for Exploring the Agriculture 4.0 Adoption

Four case studies were conducted: two in pilots of the SWAMP project (MATOPIBA pilot in Brazil and CBEC pilot in Italy); two in açaí palm farms in Brazil. The ratio of this choice was since different irrigation techniques are applied in these cases so that this exploratory study can be conducted in different conditions.

The MATOPIBA pilot is located in a region which encompasses the Brazilian states of Maranhão (MA), Tocantins (TO), Piauí (PI) and Bahia (BA), which is a critical irrigated agriculture frontier in the country. The key challenge for this pilot is to reduce energy consumption that represents up to 30% of the production costs by implementing and evaluating a smart irrigation system based on variable rate irrigation (VRI). This pilot must deal with communication instability and distance from the farm office to the central pivots. The central pivot is the irrigation technique adopted in this case. The pilot area, which alternates soybeans and cotton, is further divided into different management zones based on differences in the soil properties [7].

The CBEC (Consorzio di Bonifica Emilia Centrale) pilot is located in the Emilia-Romagna region in Northern Italy. In this pilot, three selected farms, which grow different crops (vineyards and pears) and use different irrigation techniques, are involved. The key challenges of this pilot are calculating the irrigation requirement considering the real water needs from the fields and optimizing water distribution to the farms, based on the real irrigation demand coming from the farms [7]. For this work, the pear farm is analyzed for the ease of access to the farmer and the greater interest of the farmer in experimenting with new technologies.

Two açaí farms, located in the state of Pará (Brazil), were selected for farmer interest in experimenting with advanced irrigation systems. In addition to purposive sampling, convenience sampling was adopted for the ease of access to the two farmers and their farms [84].

The questionnaire used in the case studies was based on the theory of planned behavior [19,73,85], focused on irrigation and agriculture [38], operations management [32,33,34], including questions about the challenges related to inspiration for Industry 4.0 [1,17,21,68]. The unit of analysis was the farm. The questionnaire used in case studies of açaí palm farms is illustrated in Table A2 in Appendix B.

The interviewees were the farm manager of the MATOPIBA pilot (farm manager), the farmer of the CBEC pilot (farmer A) and the farmers of the two açaí palm farms (farmer B and C). Considering the exploratory nature of the research, to reduce response bias, the definition of Agriculture 4.0 used in this work was not provided to interviewees [22,86]. Some questions were asked in different forms and using some different words, but with the same purpose, depending on whether the interviewee was an expert, a farmer of a SWAMP pilot or an açaí palm farmer. The questions based on TPB were the same in the questionnaires for expert interviews and used in the case studies of the two pilots, with the difference in the context (Agriculture 4.0 in the first, while the SWAMP project in the second). Regarding the two açaí palm farmers, instead of the word SWAMP, reference was made to sensors and drones in the questions because these are some equipment the farmer can identify as representative of innovative technologies for irrigation, as well as being equipment included in the Agriculture 4.0 context.

### 3.3. Modelling of Irrigation Operations Management

To model the agricultural operations management related to irrigation, integration definition for function modeling (IDEF0) methodology was used. IDEF0 is a modeling technique based on the Air Force Wright Aeronautical Laboratories integrated computer-aided manufacturing (ICAM) architecture for developing structured graphical representations of a system or enterprise. The use of this standard permits the construction of models comprising system functions (activities, actions, processes, operations), functional relationships and data (information or objects) within the modeled system or subject area. For new systems, IDEF0 may be used first to define the requirements and specify the functions and then to design an implementation that meets the requirements and performs the functions [87]. The main strengths of IDEF0, compared to many other functional modeling methodologies, are: simplicity, as it uses only one notational construct, called the ICOM (input–control–output–mechanism); precision, as the building of a diagram, is governed by many rules and conventions; data abstraction through a hierarchical decomposition of the system [88,89,90]. Moreover, several sources of information—interfaces—can be identified (inputs, outputs, controls and mechanisms), and management tools requirements can furthermore be pointed out [31].

**Figure 2:**
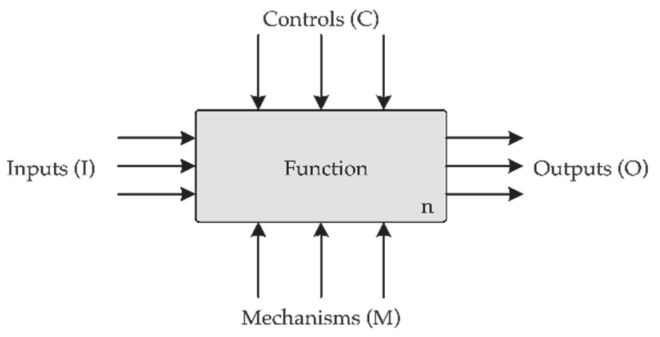
Integration definition for function modeling (IDEF0) function box and interface arrows.

An IDEF0 model consists of a hierarchical series of diagram blocks and the main components of which are box and arrow. In a diagram, shown in Figure 2, a box represents a function, and an arrow represents an interface.

An interface may be an input, an output, a control or a mechanism and is assigned a descriptive noun phrase. Inputs (I) enter the box from the left, are transformed by the function and exit the box to the right as an output (O). A control (C) enters the top of the box and influences or determines the function performed. A control is a piece of information that facilitates the execution of the function (i.e., a written procedure, an oral command, a worker experience, etc.). A mechanism (M) is a tool or resource which performs the function. Each box on the diagram may be decomposed into a lower level of detail. This feature restricts the amount of information that may be contained in the model on a single level. The resulting diagrams form a hierarchy of information that is summarized in a node tree [25].

A systematic methodology for static functional specification of system or enterprise [32], IDEF0 can be used in combination with simulation models for the support it provides in model documentation and data collection. The level of model detail has clear implications for data collection. As the model detail increases, more data may be introduced, with an impact on the development of the simulation model. IDEF0 allows a system to be described as complete a level of detail as desired [37].

### 3.4. Simulation Design

Based on the IoT sensing technologies of the weather station and satellite, shown in Table 1 and limiting to the MATOPIBA pilot context, the simulation uses irrigation water requirements estimation in line with [8,41,91]. In this study, the simulations are performed using six scenarios (named: S1.1, S1.2, S2.1, S2.2, S3.1 and S3.2) to explore the relationship between different solutions of data access and different irrigation strategies. These scenarios are resumed in Table 3.

The first scenario S1.1 consists of retrieving the reference evapotranspiration available on the study site weather station. The second scenario S2.1 consists of using the weather station climatic parameters and FAOPM model for estimating the reference evapotranspiration. Finally, in the third scenario, S3.1, the climatic parameters are obtained from a gridded weather dataset to estimate the reference evapotranspiration with the FAOPM model as well. To investigate the impact of different irrigation strategies [92] determined by the farmer behavior, for each of the three scenarios (S1.1, S2.1, S3.1), another scenario is proposed (defining S1.2, S2.2, S3.2 scenarios) taking into consideration the parameter soil saturation.

### 3.5. Data and Processing

**Figure 3:**
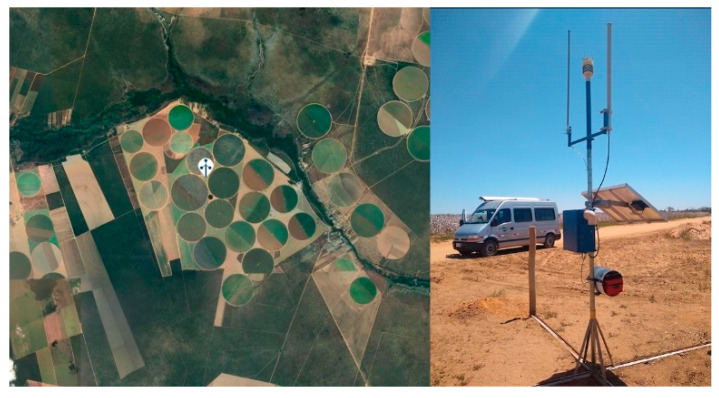
Simulations location site and weather station.

Using the MATOPIBA pilot for the simulations, data collected for this study covered 132 days of growth stages of soybean during the period of October/2019 to February/2020. In the different scenarios, weather data were obtained from the local weather station and NASA/POWER gridded weather database [57]. The geolocation of the data collected shown in Figure 3 has a latitude of 12°10′44.8″ S, the longitude of 45°31′53.4″ and an altitude of 733.5 m above sea level. The region has a savannah climate subtype [7].

The data collected from the weather station on a daily scale is maximum and minimum air temperature ($T_{max},T_{min}$), maximum and minimum air relative humidity ($RH_{max},RH_{min}$), solar radiation ($R_{a}$), wind speed at 2 m-high ($u_{2}$), precipitation (P) and reference evapotranspiration ($ET_{o}$). To test the simulation scenarios S2.1 and S3.1, the reference evapotranspiration is obtained by the FAOPM model shown in Equation (1):

(1) $$ET_{o}=\;\frac{0.408\;\Delta \;\left(R_{n}-G\right)+\gamma \;\frac{900}{T+273}\;u_{2\;}\left(e_{s}-e_{a}\right)\;}{\Delta +\gamma \left(1+0.34u_{2}\right)}$$

where $ET_{o}$ is the reference evapotranspiration in mm/day; Δ represents the slope of vapor pressure expressed in kPa/°C; $R_{n}$ is the net radiation of the hypothetical green grass crop, expressed in ${\mathrm{MJ}\;m}^{-2}{\;d}^{-1}$; γ is the psychrometric constant expressed in kPa/°C; T is the mean daily air temperature at 2 m height in °C; $u_{2\;}$ is the wind speed at 2 m height in m/s; *e_s_* is the saturation vapor pressure at 2 m height, and *e_a_* is the actual vapor pressure at 2 m height expressed in kPa.

To perform the simulation scenarios S3.1 and S3.2, weather data are retrieved from NASA/POWER gridded weather database [57]. The NASA/POWER gridded weather database uses different data sources such as NASA’s fast longwave and shortwave radiative fluxes (FLASHFlux), NASA’s modern era retro-analysis for research and applications (MERRA-2), assimilation models and GEOS, at a global 0.5° latitude and longitude grids to estimate the climatic factors [56,57]. The climatic factors collected, on a daily scale, are maximum and minimum air temperature ($T_{max},T_{min}$), mean relative humidity ($RH_{m}$), solar radiation ($R_{s}$), extraterrestrial solar radiation ($R_{a}$), clean sky solar radiation ($R_{so}$), wind speed at 2 m-high ($u_{2}$) and accumulate precipitation (P). Table 4 resumes the parameters of each IoT sensing technology.

This study uses the single crop coefficient approach for a crop of soybeans under standard conditions on all simulation scenarios. This approach combines the effects of evaporation and transpiration into one single coefficient, which is used on the determination of crop water requirements for weekly, monthly or longer periods [41,91,93,94]. The farm manager provided the growth stages used on his farm, which were associated with the crop coefficient ($k_{c}$), obtained by the FAO manual number 4 [41]. Thus, $ET_{c}$, the crop evapotranspiration, can be calculated as follows in Equation (2):

(2) $$ET_{c}=\;ET_{o}\;\times \;k_{c}$$

The irrigation water requirements were obtained using the water balance equation [91] shown in Equation (3):

(3) $$IR=ET_{c}+SAT+PERC+WL-\;P_{e}$$

where IR is the irrigation water requirements, $ET_{c}$ is the crop water requirement, SAT is the water used to saturate the soil before planting, PERC represents the percolation or seepage losses, WL represents the water layer to be maintained during the growing season, $P_{e}$ is the effective rainfall, obtained by the USDA soil conservation service method [41]. All the terms in the water balance are in millimeters. The parameters PERC and WL are considered out of scope, due to the necessity to use soil measurement devices that are not contemplated by the IoT sensing technologies used in the simulations. SAT was obtained by the farm manager and is used on the simulations S1.2, S2.2 and S3.2.

## 4. Results

This section presents the results of the coding process used for identifying the factors that can affect the adoption of PA in the context of Agriculture 4.0. The factors resulting from interviews with experts are described first, to continue with the factors resulting from case studies and with the proposition of a model of irrigation operations management using IDEF0. The section ends up with the presentation of simulation findings.

Findings from expert interviews and case studies, combined with concepts of TPB, Agriculture 4.0, PA, operations management and irrigation, are used for identifying the factors that can affect the adoption of PA in the context of Agriculture 4.0. These findings are arranged to answer the research question and coded based on the TPB predictors (attitude and perceived behavioral control), categories and factors resulting from the related work [19,23,95]. This approach is similar to the three-level codebook used by [96] to analyze the data collected through interviews, in which the three coding levels are equivalent to the three levels identified in this work: TPB predictors, categories and factors. Interview responses and case study data were classified by TPB predictors for identifying categories (e.g., “performance measures”, “access to data”, “operations planning and control”) and factors. An example of a coding process for identifying the category “performance measures” and the factors “water use” and “energy use” is shown in Table 5.

During the coding process, it was checked whether the interviewee had used the same category or factor in other answers, considering that the questions asked were semi-structured. The answers were analyzed to identify the factors to be assigned to each category (e.g., in the case of “performance measures”, the factors “water use efficiency”, “energy cost” and “water cost”) [98]. The categories and factors identified were then applied to the data collected in the case studies to carry out within-case analysis and cross-case pattern search [23]. Findings from interviews with experts and case studies were triangulated in order to allow a surplus of knowledge [22].

### 4.1. Factors That Can Affect the Adoption Resulting from Interviews with Experts

The factors resulting from expert interviews are shown in Table A3 in Appendix C, classified according to the TPB predictors and associated with the categories resulting from the related work. Table A3 presents a selection of quotes from expert interviews. The answers to the additional questions were associated with the most appropriate category, according to the three-level coding process described above. Factors not related to operations management and factors related to external actors to the farm are considered outside the scope of this work.

Performance measures, access to data, operations planning and control are the advantages of the adoption of PA in the context of Agriculture 4.0 indicated by most of the experts. Regarding performance measures, expert D stated: “In the case of precision agriculture for irrigation, the topic of cost reduction is essential, because you will work with minimizing resources and optimizing inputs”. Expert H relates water-saving to energy cost: “*If you are using less water, you are also using less energy*”. Expert I highlighted the fundamental role of water variable rate management to achieve irrigation efficiency. Experts H and G said that the use of sensors leads to access to much more data, such as evapotranspiration, soil characteristics, crop characteristics and rainfall. Expert H added that all this information could be used to calculate the irrigation water requirement.

Regarding operations planning and control, experts A, E and F suggested adopting the industrial model, which consists of the components: planning, scheduling and control. Expert A recommended scheduling tools and Gantt chart, while expert E and F highlighted the challenges of factor “weather”. Most interviewees indicated the following factors as critical: irrigation planning, weather forecast, harvest time, irrigation window, irrigation execution, irrigation control, performance indicators and farm size. Expert F mentioned the farm resources (raw material, equipment and people) involved in the operations. Experts A and E added sensors, which provide data to apply variable rate irrigation, as stated by expert I. Expert H highlighted the advantage of automation in activating the resources of the irrigation system. However, expert I stated that the factors “mobility degree of production facilities” and “coverage of agricultural field” pose challenges for operations planning and control related to farm size and connectivity infrastructure.

The main change in the adoption of PA in the context of Agriculture 4.0 entails for the farmer is the management of the rural property and agricultural operations, as declared by the experts D, G and F.

### 4.2. Factors That Can Affect the Adoption Resulting from Case Studies

The factors that can affect the adoption of PA in the context of Agriculture 4.0 resulting from case studies of açaí palm farms and pilots in Italy and Brazil are classified and presented according to the categories and factors resulting from the related work. These factors, shown in Table 6, were triangulated with findings from interviews with experts.

The farm area of the MATOPIBA pilot is 105 hectares. The source of water for irrigation is a river. The irrigation technique is the central pivot. Two pivots are supplied by electrical pumps, including the pilot pivot; the other pivots are supplied through a reservoir located in the middle of the farm. The reservoir, with a capacity of 150,000 m^3^, allows reducing evaporation (around 20%) during water distribution. Regarding the CBEC pilot, the experimental area is 1.2 hectares. Access to water occurs through Consorzio di Bonifica Centrale, which distributes water to farms through a complex infrastructure of canals and pump stations. The irrigation technique is drip irrigation. The first açaí palm farm has not yet been irrigated. A well, located about 500 m away from the cultivated area, is available for irrigation. In the second açaí palm, access to water occurs through three springs. The farmer does not currently irrigate açaí palm cultivation as it rains enough.

In the MATOPIBA pilot, the expectation of the farm manager is to optimize water use and to reduce the irrigation cost, which is mainly the electricity cost. If he can use the necessary amount of water according to the type of soil and the water holding capacity of each pivot zone, he will be able to optimize both financial and water resources. For the farmer of the CBEC pilot, the advantages are related to the possibility of achieving water savings and experimenting with new approaches based on scientific methods. The two açaí palm farmers stated that the advantages of adoption are mainly related to the possibility of reducing both the use of water and electricity. Regarding the information the farmer considers necessary to adopt sensors and drones, farmer B cited especially the daily water requirement since there are no available scientific studies to be used as guidance. It is important to know the equipment and electricity costs, according to farmer C.

Regarding irrigation planning, in the MATOPIBA pilot, the water requirement is estimated based on the weather forecast, soil moisture and field capacity, evapotranspiration (obtained through a weather station located on the farm), crop demand according to the crop stage and a manual check. The irrigation time is estimated based on the electricity cost, which defines the turning-on time (around 9:00 pm) and the turning-off time (around 6:00 am) of the pivots. In the CBEC pilot, the water requirement for irrigation is defined through the CRITERIA model, based on water balance, provided by the ARPAE regional agency, since tools for the collection of these data are not available in farms [99,100]. The irrigation time is decided by the farmer based on his experience. Regarding açaí palm farms, a scientific reference study is not available for the estimation of the irrigation requirement. All producers known to the farmers B and C adopt a requirement equal to 120 liters per day per açaí plant. Such practices came from native people from the north of Brazil and have no scientific support or further studies. When açaí crops are irrigated, farmer B and C will consider this requirement as a reference.

As for irrigation control, the farm manager of the MATOPIBA pilot manually analyzes the soil moisture. He tries to perform night irrigation, which is cheaper (at night, irrigation costs are 10 times lower than during the day). He stated: “Night irrigation is what makes the business feasible”. The pivot control system generates reports and indicators to evaluate the efficiency of irrigation. The Italian farmer controls the irrigation exclusively by his own experience. Both farmers B and C use the experience and information shared with other farmers to control visually and manually the irrigation.

For the farm manager from MATOPIBA pilot, the main changes are managerial, in terms of training to learn about the new tools and to know how to read the data correctly, to support the decision-making process. In this pilot, the main challenge is the decision about where to irrigate, based on IoT sensing technology of soil probe and therefore implement variable rate irrigation. This allows zone management, which must be described in the geographic information system, in order to obtain the water requirement in the form of an irrigation prescription map. The key challenge of the CBEC pilot is to calculate the irrigation requirement through the use of the CRITERIA model considering the real requests from the field, according to a water balance approach. The adoption of UAV is being tested to check the model estimation and to refine the plant parameters. The use of a multispectral camera allows collecting data fundamental to calculate the NDVI and the LAI [39,54,55]. Therefore, the LAI estimation, currently based on literature data, will be improved, thanks to the data collected through the UAV. As for the two açaí palm farms, both farmers B and C are involved in an ongoing irrigation project for açaí cultivation and are interested in experimenting with innovative technologies for irrigation.

### 4.3. Modelling of Irrigation Operations Management

**Figure 4:**
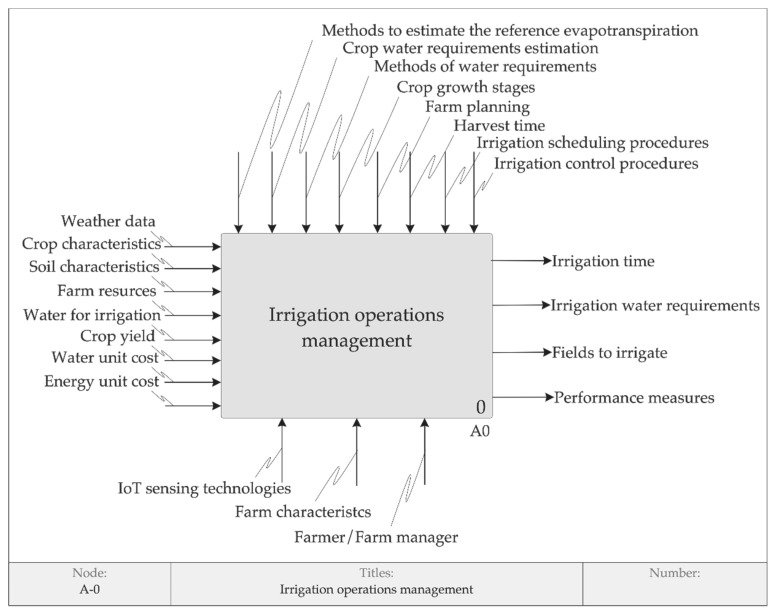
Context diagram of irrigation operations management.

As a result of the factors identified whit the related work, expert interviews and case studies, a model of irrigation operations management through IDEF0 is proposed, combining concepts of IoT, PA, Agriculture 4.0, TPB, irrigation and operations management. This model, built using the approach proposed by [87], supports the formulation of the simulation scenarios and the development of theory on the adoption of PA in the context of Agriculture 4.0. The context diagram of irrigation operations management, shown in Figure 4, depicts the top-level function being modeled and its inputs, controls, outputs and mechanisms.

**Figure 5:**
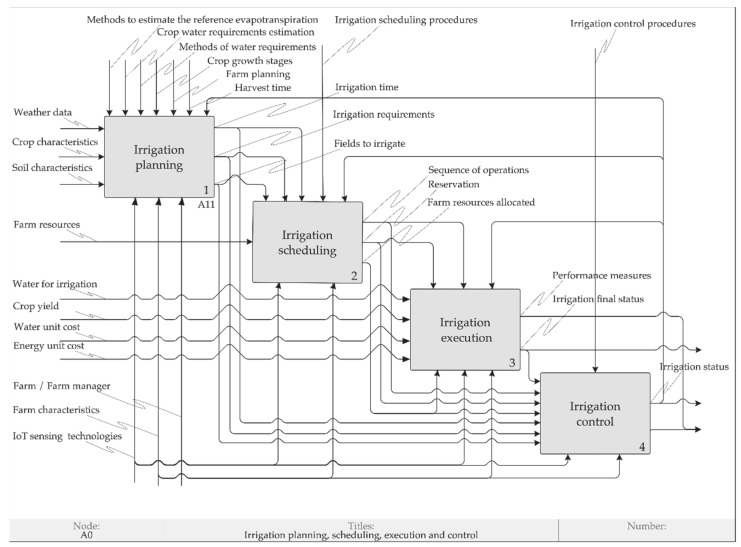
IDEF0 diagram of irrigation operations management.

The context diagram is decomposed into four functions, as shown in Figure 5: irrigation planning, irrigation scheduling, irrigation execution and irrigation control. This model differentiates planning and scheduling in line with the literature on IDEF0 [32,33,34] and the suggestion by experts A, E and F.

The irrigation planning produces the irrigation water requirements [39,41,55,91], the irrigation time [101] and the fields to irrigate [102]. Farm planning involves several seasonal decisions that the farmer must make, such as determining the crops to be grown, the area to be used for each crop, the irrigation policy [103], including irrigation strategies [92]. The seasonal nature of the operations is one of the main characteristics that differentiate agriculture from industry, as highlighted by the expert B: “*We work in the agricultural cycle, you have the right time to plant and if you make a mistake, you have committed a whole harvest. Agriculture is a window, so this must be done before, based on this window*”. The inputs transformed by irrigation planning are crop characteristics, weather data and soil characteristics [9,39,41,91,104,105]. The resources which perform the irrigation planning are farmer or farm manager (depending on farm organization), farm characteristics and IoT sensing technologies [9,39,52]. The farmer or farm manager supervises all operations. The fundamental role of resources is highlighted by the expert F: “*For the planning of agricultural operations, the following information is required: the necessary resources at the level of inputs (i.e., raw materials), the hardware (i.e., what equipment you need), the resources of people (i.e., how many people are involved in this process)*”.

The irrigation scheduling defines which farm resources must be allocated. Farm resources to be allocated are men and machinery [106] and irrigation system resources, which depend on farm characteristics and crop type, such as valves and pumps [101,102,107], central pivots [102], river, well, spring, canals, reservoir, drip irrigation system). As an example, the farm manager of the MATOPIBA pilot explained: “*In the farm, there is a river, which is the water source for the farm irrigation. The two nearest pivots are supplied by electrical pumps, including the pilot pivot. The other pivots are supplied through a reservoir located in the middle of the farm. The water is taken by electrical pumps to this reservoir, which distributes the water to the other more distant pivots*”. The irrigation scheduling also produces a reservation in the tuple < *operation*, *time window*, *resources* > and a sequence of the operations to be performed [68]. An example can be illustrated with the MATOPIBA pilot, in which the operation “irrigation execution”, using the resource “central pivot 8”, is carried out in the time window “beginning at 21:00 h on day 1 and ending at 6:00 h on day 2”. Irrigation scheduling procedures can include which crop should receive priority when allocating water during the next irrigation turn [108], priority rules based on management objectives [34]. An example of a priority rule is the minimization of the energy cost compared to water use. In the case of the MATOPIBA pilot, this rule determines the nightly execution of the irrigation. Another example is the possibility of a switch on/off a pump/valve when the water level applied to the field reaches some predefined threshold value [101].

The irrigation execution concerns the realization of scheduled operations [68,109]. Activating the scheduled resources in the context of Agriculture 4.0 is automatic, according to the expert H: “*The entire process is done automatically, which reduces labor work. You don’t have to turn on the pivot, you don’t have to turn on the irrigation system under the surface of the soil*”. Water for irrigation can be considered unlimited and limited, depending on the amount of water available for irrigation or the capacity of the water distribution system [110]. The irrigation execution outputs are the final irrigation status and the following performance measures: water use, water use efficiency, water cost and energy cost. The water use efficiency, which can be defined and measured in different ways [111], is influenced by several factors, such as the timing and the quantity of water applied during irrigation, the losses along with the water distribution and the type of irrigation technique [40].

Irrigation control receives, as inputs, the outputs from the other functions and the control commands from resources allocated [10,107,112,113]. Irrigation control monitors the irrigation system and resources allocated, through their identification, operation, location and status [10], based on irrigation control procedures. This possibility of controlling irrigation operations through IoT sensing technologies, like soil probes, can affect Agriculture 4.0 adoption, as stated by expert F: “*The control would be much easier thanks to the use of sensors, in the context of IoT for irrigation, for example checking if that plant needs water, nutrients*”. This will improve the visual inspection based on experience carried out by farmers A, B and C. Moreover, expert B added that the adoption of PA in the context of Agriculture 4.0 would allow monitoring exactly how much water, where, in which way, at what time, in what location. This control will overcome the risks of under and over-irrigation, as reported by [9]. Irrigation control monitors irrigation status, based on information received from planning, scheduling and execution operations, to provide feedback to the other functions [34,87,114]. Irrigation control also allows calculating performance measures, in addition to results directly related to the use of water and energy provided by the irrigation execution. Like in the manufacturing sector, agricultural farms should initiate key performance indicators for monitoring and reviewing the performance [97]. This is highlighted by the expert F: “*Traditional industrial indicators, such as efficiency, productivity, capacity, availability, can be calculated and used, as in factory operations*”. Expert G added: “*I believe that in the future the movement 4.0 will bring some close developments, for example, greater reliability, greater precision, greater productivity and, an issue that is very present in the industry, greater traceability*”. Irrigation control procedures concern control information for carrying out irrigation operations. An example of the procedure concerns the irrigation water requirement and irrigation time. In line with the industrial model, if the actual values of these planned outputs deviate from the expected value, feedback is sent to the function irrigation planning [32,115] for reevaluating the existing irrigation programs [110]. Another example involves the information for maintaining the soil moisture content between the field capacity and the permanent wilting point, in the case of IoT sensing technology of soil probe [104].

### 4.4. Simulations: Application of the Model of Irrigation Operations Management

**Figure 6:**
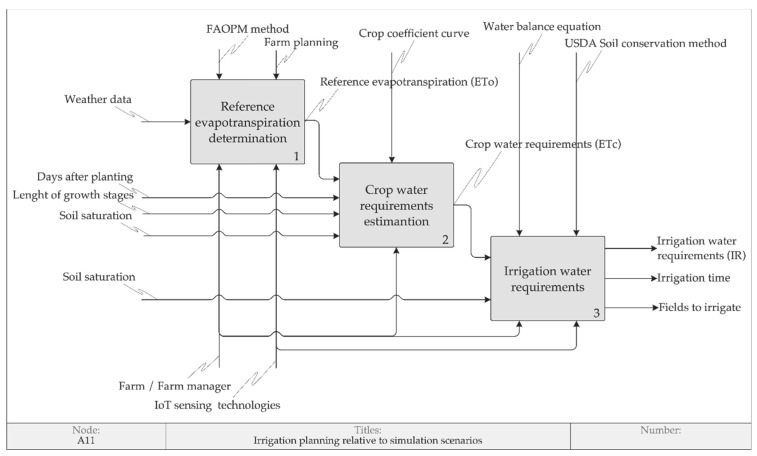
IDEF0 diagram of irrigation planning relative to simulation scenarios.

The proposed model of irrigation operations management provides an initial framework to study the identified factors, the relationships among them and, consequently, the adoption of PA in the context of Agriculture 4.0. The first application of this framework aimed to evaluate the cause–effect relationships among some factors involved in irrigation planning, is presented in this section, which illustrates the results of the simulations performed with MATOPIBA pilot, considering the crop type soybean and the “central pivot 8”. These simulations use the IDEF0 modeling shown in Figure 6.

Using the methods proposed by [8,41,91], the irrigation planning can be decomposed into three operations: reference evapotranspiration determination, crop water requirements estimation and irrigation water requirements estimation been represented by the respective IDEF0 functions.

The reference evapotranspiration determination produces the $ET_{o}$ parameter. On the MATOBIBA pilot, this parameter is obtained from the local weather station. In addition, two new reference evapotranspiration estimations are studied: the $ET_{o}$ derived from local weather station weather data as input and the FAOPM model; the $ET_{o}$ derived from IoT sensing technology of satellite estimated weather data as input and the FAOPM model.

The crop water requirements estimation transforms the inputs of reference evapotranspiration, days after planting, length of growth stages and tabulated coefficients from the local 132 days soybeans crop, using the crop coefficient curve, provided by [41], to produce the crop water requirements along the growth stages.

**Figure 7:**
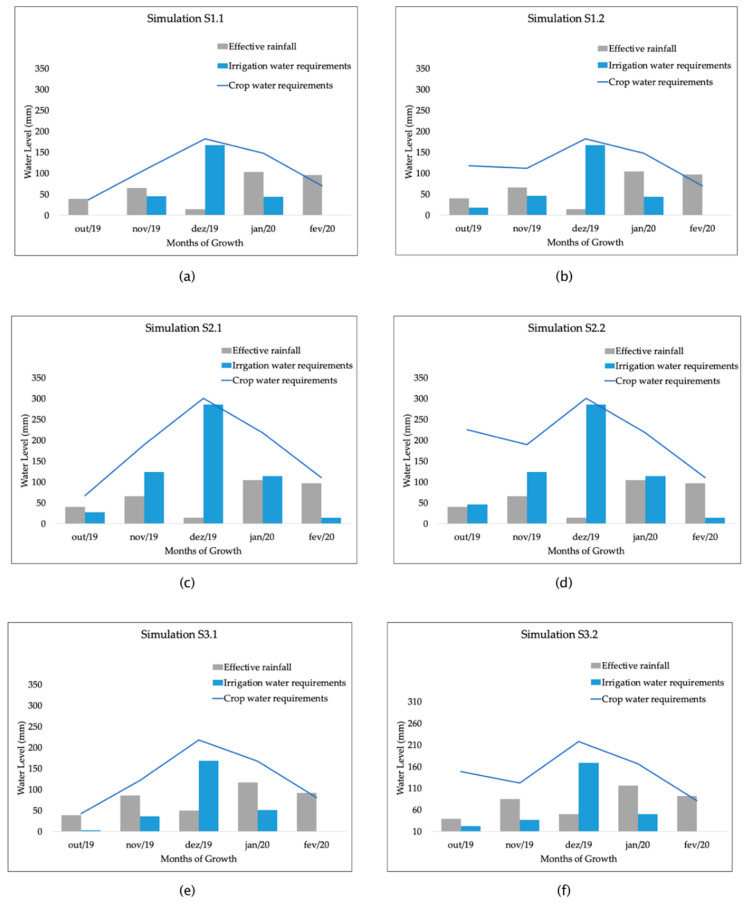
Results of the simulations performed. (**a**) Results of Simulation S1.1; (**b**) Results of Simulation S1.2; (**c**) Results of Simulation S2.1; (**d**) Results of Simulation S2.2; (**e**) Results of Simulation S3.1; (**f**) Results of Simulation S3.2.

The third operation produces irrigation requirements (IR), based on the USDA soil conservation service method and the water balance equation. The operation took as inputs the $ET_{c}$, weather data of rainfall and the soil saturation water (SAT). As highlighted by the farm manager: “the saturation parameter is used to reach the field capacity, which makes available water for seedlings on the initial growth stage”. Figure 7 and Table 7 show the results of the simulation scenarios.

#### 4.4.1. Simulations of Scenarios S1.1 and S1.2

The first simulation (S1.1) performed uses the reference evapotranspiration provided by the weather station as well as the crop coefficients of soybeans to produce the crop water requirements (using Equation (2)). The effective rainfall from the weather station is used to produce irrigation water needs (using Equation (3)).

In the charts in Figure 7a, irrigation water requirements follow the variability of rainfall and crop water requirements on each growth stage. Despite this, the irrigation recommended in the first and last stages was 0 mm. The results on these stages can be explained by the low crop water requirements, which is supplied by the high amount of effective rainfall.

The simulation considering the SAT parameter (S1.2), shown in the chart in Figure 7b, had an increase of 6.81% in the total amount of water used on the initial growth stage. The chart in Figure 7b shows that the crop water requirements chart represents the soil evaporation in the stage before sowing and has a decrease when the curve embodies the crop water requirements at the beginning of the first stage in the first month of growing.

#### 4.4.2. Simulations of Scenarios S2.1 and S2.2

On the simulations S2.1 and S2.2, the reference evapotranspiration is determined using the weather data from the local water station (using Equation (1)) and the crop coefficients of soybeans to produce the crop water requirements (using Equation (2)). The irrigation water requirements are obtained through effective rainfall and crop water requirements (using Equation (3)).

The results shown in the chart in Figure 7c reveal an increase of 119.93% of the total amount of water used in irrigation, compared with the S1.1 simulation, where no irrigation water is used in the initial and final stages. This is explained with the reference evapotranspiration parameter, which had its values increased by 61.51%, compared with the simulation S1.1. The rising of the reference evapotranspiration leads to an increment in crop water requirements. Once the precipitation does not supply the increase in crop water requirements, it will lead to more irrigation water.

The simulation S2.2, which uses the SAT parameter, increased 3.10% compared with the S2.1 simulation in the total amount of irrigation water. The following stages had the same behavior variability of S2.1 simulation.

#### 4.4.3. Simulations of Scenarios S3.1 and S3.2

The simulation S3.1 uses the climatic parameters obtained from the satellite estimated weather data to determine the reference evapotranspiration (using Equation (1)) and the crop water requirements (using Equation (2)). Using the effective rainfall from the same IoT sensing technology, the irrigation water requirements are determined (using Equation (3)).

The results of the S3.1 simulation is close to those of the S1.1, as shown in Table 7, in which a difference of 0.96 mm of the total amount of irrigation water was found. The chart in Figure 7e shows that the variation of the parameters measured and IoT sensing technologies adopted contributes to the variability of the chart variability of reference evapotranspiration and crop water requirements, in which a difference of 180.05 mm and 256.08 mm, respectively, was found.

The results of simulations 3.1 and 3.2, shown in charts (e) and (f) of Figure 7, also highlight that the rainfall is overestimated when compared with the precipitation obtained from the weather station on simulations S1.1, S1.2, S2.1 and S2.2. The simulation S3.2 uses 7.33% more water than the simulation S3.1. Water used in the stage before sowing presents a result slightly higher than simulations S1.2 and S2.2, due to the overestimation of precipitation in that period. However, the simulations S3.1 and S3.2 have the same variability along the crop growth stages.

## 5. Discussion

Within this section, the results and their contributions are discussed, starting with relevant factors for the adoption of PA in the context of Agriculture 4.0. Afterward, the proposed model of irrigation operations management based on IDEF0 and the results of simulations are discussed. Finally, limitations of the study and future research are presented.

### 5.1. Relevant Factors for the Adoption of PA in the Context of Agriculture 4.0 and the Key Role of IoT

This work contributes to current knowledge on Agriculture 4.0, proposing a list of categories and factors that can affect the adoption of PA in the context of Agriculture 4.0 through empirical research. The main categories that drive the adoption of PA in the context of Agriculture 4.0, focusing on farmer behavior and operations management are performance measurement, access to data, operations planning and control, farm characteristics. These categories and related factors, such as water use, energy cost, irrigation water requirement, irrigation planning, irrigation control, can influence the perception of the farmer in the evaluation of the investigated behavior and therefore impact his intention to adopt it. In line with [19], the domain-specific factors are important to gain a complete understanding of the behavior under consideration. In this study, these factors are: farm size, methods to estimate the reference evapotranspiration, methods to crop water requirement estimation, crop characteristics, weather data, soil characteristics, farm resources, crop growth stages, farm planning, crop yield, water use efficiency, irrigation planning, irrigation control, in the agriculture domain; scheduling, resources to be allocated, farm resources allocated, reservation, the sequence of operations, performance measures, in the operations management domain; IoT sensing technologies and parameters measured through these technologies, in the IoT domain.

The greater the perception of the farmer regarding the benefits he can obtain (such as reduction of water use, reduction of energy costs, improvement of decision-making process relating to irrigation planning, scheduling and control), the more favorable the attitude of the farmer and consequently stronger his intention to adopt the PA in the context of Agriculture 4.0. Furthermore, the lower the perception of the farmer concerning obstacles, such as competency in managing the agricultural operations as reported by many experts, and the more resources the farmer believes he possesses, like IoT sensing technologies, the greater is his perceived control and consequently stronger his intention to adopt the PA in the context of Agriculture 4.0.

This work contributes to the progress of knowledge on Agriculture 4.0, proposing a list of factors related to farmer behavior and operations management, as a result of empirical research in four farms with different size, organization and irrigation technique, and overcoming the limit that Agriculture 4.0 is still restricted and put off in theory [15]. The empirical findings contribute to answering the suggestion of [16] regarding a more in-depth analysis needed to understand what Agriculture 4.0 is and also to include the perspective of operations management in the Agriculture 4.0 definition. In line with the definition of precision agriculture [48], the definition of IoT of [116], the definition of Agriculture 4.0 formulated by [16], shown in Table 8, and the IoT sensing technologies presented in Table 1, the Agriculture 4.0 in this work is:

“*a management strategy, evolution of precision agriculture, realized through the automated collection, integration and analysis of temporal, spatial and individual data, collected by IoT sensing technologies and farm resources, making in this way possible the generation of knowledge, to support the design of applications for the farmer decision-making process in irrigation operations management*”.

The interconnection of IoT sensing technologies (such as satellite databases, weather station, soil probes, UAV) and farm resources (such as pumps, central pivots and valves) supports the design of applications, like an estimation of crop water requirement, allocation of farm resources, the definition of irrigation operations sequence, control of irrigation status and calculation of performance measures. These applications allow the farmer to make decisions concerning planning, scheduling, execution and control of irrigation. This definition highlights the key role of IoT in the evolution of precision agriculture towards Agriculture 4.0.

### 5.2. Proposed Model of Irrigation Operations Management

The identified factors allow to understand the management of irrigation operations and formalize it in a model, using the IDEF0 methodology. Considering the lack of theory on Agriculture 4.0 [15,16], this model contributes to the current level of knowledge on Agriculture 4.0, proposing relationships among those factors in order to explain the adoption of precision agriculture in the context of Agriculture 4.0. These relationships were identified during the construction of the model, decomposed into four functions illustrated in Figure 5: irrigation planning, irrigation scheduling, irrigation execution and irrigation control.

In contrast to previous research on agriculture, the proposed model distinguishes between planning and scheduling. Considering terminological aspects [33], planning and scheduling can have a different meaning in the literature. Some authors use irrigation scheduling to indicate how much irrigation water has to be given to the crop and how often or when this water is given [91], to meet a specified management objective [112] and based on different approaches (e.g., soil water measurement, soil water balance calculations, plant stress sensing) [117]. Some authors use irrigation planning for referring to the planning horizon. According to [110], irrigation planning refers to seasonal irrigation, with irrigation execution in terms of days since planting time. [118] refer to short-term planning (7–14 days) for the estimation of irrigation water demand and rainfall in the irrigation season. [119] differentiate between irrigation planning over a time horizon that is usually long and a water distribution schedule that is usually daily. Recently [68] introduced a differentiation in agricultural operations management about planning and scheduling. The planning determines which operations to execute and with what resources, while the scheduling determines the times for executing the selected operations.

In contrast, in a manufacturing system, the planning involves the determination of the type and amount of products to be produced in a future time frame, while the scheduling involves the allocation of operations to the resources along with the specific start and finish times in each period [32]. The allocation decisions for the production resources are the primary focus of the production scheduling [120]. Therefore, the proposed model distinguishes between irrigation planning and irrigation scheduling in line with the literature on IDEF0 [32,33,34] and the quotes of experts A, E and F. This model complements the recent work of [68] who do not concentrate their study on irrigation operations management. The framework of the four-function model highlights the resources involved in each function, their role in performing the operations, the interfaces between the functions in terms of inputs and outputs, the rules governing the execution of operations. An example to illustrate this difference is the MATOPIBA pilot. As a result of the simulation illustrated in the chart of Figure 7a, the farm manager receives the information that the irrigation time, relative to the “central pivot 8” studied, is equal to November and December of 2019 and January of 2020. Regarding the irrigation scheduling, the function depicted in Figure 5, on the first day of November 2019, informs the farm manager that the “central pivot 8” will be activated in the time window “beginning at 21:00 on the first day of November 2019 and ending at 6:00 on the second day of November 2019, based on irrigation scheduling procedures. This scheduling is replicated for all the days in the irrigation time.

The model of irrigation operations management also answers to the suggestion of [21], as the analysis carried out can facilitate the establishment of operational solutions to explain the unexplored potential of PA in agricultural operations. The inspiration for Industry 4.0 led to consider some advantages that can be introduced in agriculture, such as improvements in planning and control, which is related to one of the key categories (operations planning and control) of the proposed model. In addition, the formulation of the model includes the factors associated with the challenges analyzed in Section 2.3. Regarding the interaction between agricultural field operations and biologically active systems [21], the sensing technologies allow monitoring the development of the crop along the growth stages, thus mitigating the risk and uncertainty relating to operating in a biologically active environment. In this way, the farmer will be able to make decisions, also considering this challenging factor. Data collected by IoT sensing technologies of weather station and satellite can be used to support the farmer decision-making process challenged by uncertainty and risk relating to operating in a biologically active environment, as shown during simulations carried out. As for the mobility degree of production facilities [17], this factor seems to be not challenging in irrigation planning, but its influence on irrigation execution and irrigation control should be investigated in future research, as well as the availability and bandwidth of wireless connections.

The exploration of the adoption of PA in the context of Agriculture 4.0 of this work leads to expand the possibilities of the IoT in the domain of PA since the IoT can also be studied concerning the operation’s management. The availability and adoption of different IoT sensing technologies allow managing irrigation operations in terms of access to data, planning and control of operations, performance measures, compared with the current management in the farms studied. Furthermore, the focus on farmer behavior and on his perceptions, related to the advantages and disadvantages of adopting these IoT sensing technologies, allows developing these possibilities according to farmer needs.

### 5.3. Relationships Among Identified Factors in the Proposed Model

The simulation results evidence the relationships among some factors involved in irrigation planning and, therefore, to study the parameters related to these factors. These findings contribute to the development of the theory on Agriculture 4.0 and to a better understanding of what Agriculture 4.0 is [15,16].

The simulation investigates the relationship among some factors, shown in Figure 5, encoded in the categories “access to data” (crop characteristics, weather data and soil characteristics), “operations planning and control” (irrigation planning, irrigation time, irrigation water requirement, farm resources, IoT sensing technologies). The simulation findings explain the cause–effect relationships among the parameters related to these factors and the irrigation planning outputs.

In line with the literature [39,42,44], reference evapotranspiration plays an essential role in irrigation planning. The simulations results suggest that IoT sensing technologies of the weather station and satellite with different measured parameters, as shown in Table 7, affect the results of the FAOPM model in the same geolocation, changing both the crop water requirements and irrigation water needs variations.

As stated by authors as [42,44,121,122], the application of the FAOPM model using more measured parameters can lead to best performances in the determination of reference evapotranspiration compared with calculation with estimated parameters. This has an impact on the benefits perceived by the farmer in the use of these models [75] and consequently has an impact on his intention to adopt Agriculture 4.0. However, the FAOPM model, using more measured parameters, can lead to the challenge highlighted by the expert L: “*The planning of operations is a complex process and the more data the farmer has, the more he needs to set up a process with less error*”. This challenge is reported by [18], who states that data by its nature in an Agriculture 4.0 scenario become complex to manage both in terms of size and complexity of the analysis to be carried out. This challenge, however, requires future research to study in greater depth the relationships between the factors involved. Notwithstanding, researchers as [121,122] elaborate that temperature and radiation are promising parameters to estimate de reference evapotranspiration. However, new models must be studied to perform a better or equal precision of the FAOPM and to investigate the relative impact on the adoption of precision agriculture in the context of Agriculture 4.0.

The sensibility of satellite-based solutions have to cloud levels [55,123,124], and the questionable results of the use of air humidity, wind speed, and precipitation retrieved from the NASA/POWER gridded weather dataset [56] suggest some disadvantages of satellite-based solutions. However, gridded weather databases provide applicable temperature and radiation parameters [56], making room for future research of models that combine IoT sensing technologies shown in Table 1 and the proposed model of irrigation planning. Another advantage related to IoT sensing technology of satellite is its applicability to irrigation planning applications [125] and its high spatial resolution compared with the weather stations [39] been suitable to small farmers, such as the two açaí palm producers. Thus, future research is needed to investigate the adoption of IoT sensing technology of satellite in regions with high cloudiness like Pará in Brazil.

This work also contributes to Agriculture 4.0 discussion, since the possibility the farmer must use different methods to estimate the reference evapotranspiration and the crop water requirement and to adopt IoT sensing technologies that improve access to data and the operations planning, in line with literature on Agriculture 4.0 [17]. Consequently, Agriculture 4.0 enables the farmer to strengthen the decision-making process transforming the traditional operating model, often experience-based, to a digital data-intensive one [69]. Decision-making mechanism is expected to be a complex mix of human and computer factors in the future [126], mitigating the challenge posed by Agriculture 4.0 to farmers which lies in the interaction with ICT without necessarily being experts in the digital domain, but used to “understand” the crop behavior by experience and simple tools [127].

### 5.4. Limitations and Further Research

The simulation findings represent the first application of the proposed list of factors and model of irrigation operations management, depicted in this work. However, some limitations should be considered and addressed in future studies, considering the exploratory nature of this study.

This work, carried out as part of the SWAMP project, involved the MATOPIBA pilot in Brazil and one of the three farms of the CBEC pilot. Moreover, interviews with experts were conducted in Brazil. To increase the external validity of the results obtained, the multimethod approach of this study will be applied to the other farms involved in the SWAMP project, and interviews will also be conducted with experts in Italy.

Regarding the relevant factors for the adoption of precision agriculture in the context of Agriculture 4.0, the TPB predictor “subjective norm” was considered out of scope. Future research is necessary to include this predictor in the exploration and to investigate the relationship between the actors operating in the farm ecosystem (for example, the Consorzio di Bonifica Centrale in the Italian pilot) and the farmer. Future research will also focus on studying the factors identified through semi-structured interviews and considered out of scope (such as access to the market of technologies, access to the market of financial resources and access to the market of education) and their impact on the adoption. To a holistic insight, the system dynamics simulation technique will also be applied.

The model of irrigation operations management is proposed as a framework for directing future studies aimed at investigating the cause–effect relationships among the parameters relative to the identified factors involved in the adoption of PA in the context of Agriculture 4.0. Therefore, a research topic will be promoted to develop a theory on Agriculture 4.0, which in the future will be extended to other agricultural operations in addition to irrigation. Since the IDEF0 methodology is also used for the design of new systems, the proposed model will guide the design of a support system for irrigation planning, scheduling and control.

Regarding the relationships among the identified factors in the proposed model, simulations were carried out to compare the IoT sensing technologies of weather station and satellite. To choose which is the best method for estimating the irrigation water requirement, it is necessary to study the relationship also with crop yield, as well as with the water use. Therefore, future research will be carried out through field experiments, considering IoT sensing technologies, such as soil probes and UAV and other crop types, also in order to evaluate the impact of temporal and spatial variability on the adoption.

## 6. Conclusions

The aims of this work were to explore the factors that can affect the adoption of precision agriculture in the context of Agriculture 4.0, proposing a model to understand and formalize agricultural operations management based on identified factors and performing simulation scenarios to study the relationships among the identified factors. Some research gaps were identified following the related work. To address these gaps, a multimethod approach consisting of interviews with experts, case studies, modeling techniques and simulation was adopted. Findings from empirical research supported the identification of a list of categories and factors that can affect the adoption of PA in the context of Agriculture 4.0, the formalization of these categories and factors in a model of irrigation operations management, the execution of the simulation scenarios to study the relationships among some factors involved in irrigation planning.

Considering the current development of Agriculture 4.0 concepts, this work makes a theoretical contribution, proposing a list of factors related to farmer behavior and operations management that drive the adoption of PA in the context of Agriculture 4.0. Furthermore, this multidisciplinary research, bringing together the different perspectives of precision agriculture, IoT and operations management, complements the extant literature on Agriculture 4.0, mainly focused on automation, robotics and sustainability.

This research also makes a contribution to Agriculture 4.0 discussion since the possibility the farmer must use different methods to estimate the reference evapotranspiration and the crop water requirement and to adopt several IoT sensing technologies allows to improve access to data and the irrigation planning, highlighting the key role of IoT in the evolution of the precision agriculture towards the Agriculture 4.0.

The model of irrigation operations management is proposed as a framework for directing future studies, involving the other farms of the SWAMP project, the factors considered out of scope, other agricultural operations in addition to irrigation, other crop types and the design of a support system for irrigation planning, scheduling, execution and control.

## Figures and Tables

**Table 1:** Parameters measured through the Internet of things (IoT) sensing technologies.

Factors	Weather Station	Satellite	Soil Probe	UAV	References
Crop factors		Vegetation indexes, canopy temperature, leaf area index (LAI) *, surface albedo *, crop water requirements *	Water height (paddy areas), leaf-wetness	Canopy temperature, vegetation indexes, leaf-wetness *, size of crops *, shape of crops *, thickness * of the plant stem, crop coverage *, crop transpiration *,	[39,51,52,53,54,55]
Climatic factors	Rainfall, air temperature, air humidity, wind speed, wind direction, solar radiation, barometric pressure, light intensity, relative humidity	Weather forecast *, solar radiation *, wind speed *, instantaneous evapotranspiration *, air temperature *, humidity *, incoming shortwave *, radiation *, incoming longwave * radiation *, rainfall *, latent heat flux *,	Environmental temperature, environmental air humidity light intensity, solar radiation, carbon dioxide	environmental temperature, environmental air humidity	[39,51,52,55,56,57]
Soil factors		Soil surface temperature *	Soil temperature in depths, leaf-wetness, soil moisture, electrical conductivity, salinity, pH value	Soil surface temperature *, soil color, soil transpiration *	[9,51,54,55,58]

(*) Parameters measured indirectly (directly when IoT sensing technologies are used to collect the parameter, indirectly when models are used to estimate the parameter).

**Table 2:** Expert profiles.

Experts	Expert Profiles
Expert A	Agronomist, researcher, more than 20 years of experience in agribusiness
Expert B	Agronomist, researcher, more than 20 years of experience in agribusiness
Expert C	Economist, researcher, more than 20 years of experience in agribusiness
Expert D	Agronomist, researcher, more than 20 years of experience in agribusiness
Expert E	Engineer, researcher, 15 years of experience in operations management
Expert F	Engineer, researcher, more than 20 years of experience in operations management
Expert G	Agronomist, researcher, 10 years of experience in agribusiness and supply chain management
Expert H	Agronomist, researcher, 5 years of experience in agribusiness
Expert I	Agronomist, researcher, 10 years of experience in precision agriculture
Expert L	Engineer, researcher, more than 20 years of experience in precision agriculture

**Table 3:** Simulation scenarios.

Irrigation Strategies	IoT Sensing Technologies and ET_0_ Estimation		
	ET_0_ Weather Station	Weather Station Weather Data and ET_0_ FAOPM	Satellite Weather Data and ET_0_ FAOPM
Without soil saturation parameter	S1.1	S2.1	S3.1
With soil saturation parameter	S1.2	S2.2	S3.2

**Table 4:** Summary of climatic factors of each IoT sensing technology.

IoT Sensing Technologies	Temperature Parameters	Radiation Parameters	Humidity Parameters	Wind Speed Parameters	Evapotranspiration Parameters
Weather Station	$T_{max},T_{min}$	$R_{s}$	$RH_{max},RH_{min}$	$u_{2}$	$ET_{o}$ *
Satellite	$T_{max}\;**,T_{min}\;**$	$R_{s}\;**$,$\;R_{a}\;**,\;R_{so}\;**$	$RH_{m}\;**$	$u_{2}$ **	

(*) Parameter estimated from the local weather station. (**) parameter estimated from NASA/POWER gridded weather database.

**Table 5:** Example of a coding process for identifying categories and factors.

TPB Predictor	Related Work	Quotes from Interviews	Category-Factors
Attitude (advantages, benefits, expected positive results)	Like the manufacturing sector, agricultural farms should initiate key performance indicators for monitoring and reviewing the performance [97]. Industrial production takes place in close, well-defined environments in which performance data can be measured by deterministic matters [21]. Definition of water amount to be applied and irrigation time have a direct effect on the water use efficiency [40].	“The Agriculture 4.0 adoption will allow knowing exactly how much water, where, in which way, at what time, in what location. Moreover, with another important factor, the cost of energy”, Expert B. “I know the possibility of controlling water use using sensors, which can be useful to reduce water and electricity use”, Farmer A.	Performance measures—Water use, energy use

**Table 6:** Factors that can affect the adoption resulting from case studies.

Categories	Factors	MATOPIBA Pilot Farm	CBEC Pilot Farm	Açaí Palm Farm 1	Açaí Palm Farm 2
Farm characteristics	Crop types	Soybean, corn, sorghum and cotton	Pear	Açaí palm, piper nigrum	Açaí palm, cocoa
	Farm size	912 hectares (696 irrigated), 7 central pivots	21 hectares	225 hectares (açaí palm cultivation: 20 hectares)	100 hectares (açaí palm cultivation: 70 hectares)
	Distance shed-cultivated fields	Nearest pivot at 700 m, farthest pivot at 4 kilometers	Shed close to cultivated fields	700 m	Shed in middle of farm
	Internet	4G	4G	3G	3G
Performance measures	Expected savings	Energy cost, water use, management zones	Water use, the scientific method	Water use, energy cost	Water use, energy cost
Operations planning and control	Farm resources	16 farm workers, river, electrical pumps, reservoir, central pivots	Canal, electrical pump, drip irrigation	3 farm workers, well, an irrigation system in progress	5 farm workers, springs, an irrigation system in progress
	Irrigation planning	Scientific method	Scientific method and experience	Experience	Experience
	Irrigation control	Mechanical and manual	Visual and manual	Visual and manual	Visual and manual
Changes	Irrigation projects	In progress	In progress	In progress (açaí palm)	In progress (açaí palm)
	IoT sensing technologies	Soil probe (in progress)	UAV (in progress)	Interest in experimenting	Interest in experimenting

**Table 7:** Results of the simulations performed.

Irrigation Planning Outputs	Simulation 1		Simulation 2		Simulation 3	
	S1.1	S1.2	S2.1	S2.2	S3.1	S3.2
Reference evapotranspiration determination (${\mathrm{ET}}_{o}$/mm)	628.75	709.05	1036.87	1194.40	856.61	962.48
Crop water requirements (${\mathrm{ET}}_{c}$/mm)	548.13	628.43	885.31	1042.74	629.23	735.10
Irrigation water requirements (IR/mm)	257.27	274.80	565.81	583.34	258.22	277.15

**Table 8:** Definitions of precision agriculture (PA), IoT and Agriculture 4.0.

Definition of PA [48]	Definition of IoT [116]	Definition of Agriculture 4.0 [16]
Precision agriculture is a management strategy that gathers, processes and analyzes temporal, spatial and individual data and combines it with other information to support management decisions according to estimated variability for improved resource use efficiency, productivity, quality, profitability and sustainability of agricultural production.	Internet of things is a conceptual framework that leverages the availability of heterogeneous devices and interconnection solutions, as well as augmented physical objects providing a shared information base on a global scale, to support the design of applications involving at the same virtual level both people and representations of objects.	Agriculture 4.0 is the evolution of precision agriculture, realized through the automated collection, integration and analysis of previously separated data silos coming from the field, equipment sensors and other third-party sources, enabled by the use of smart and digital technologies of Industry 4.0, making in this way possible the generation of knowledge, to support the farmer in the decision-making process in the farm enterprise.

## Appendix A. Questionnaire Used in Expert Interviews

**Table A1:** Questionnaire used in expert interviews.

**Opening Question**	What do you think about Agriculture 4.0?
**TPB Predictors**	**Questions**
	What are the advantages of the adoption of precision agriculture for irrigation in the context of Agriculture 4.0?
Attitude	What are the disadvantages of the adoption of PA for irrigation in the context of Agriculture 4.0?
	What do you think of the changes a farmer needs to make to adopt the PA for irrigation in the context of Agriculture 4.0?
Perceived behavioral control	What factors would make it easy for a farmer to adopt the PA for irrigation in the context of Agriculture 4.0?
	What factors would make it difficult for a farmer to adopt the PA for irrigation in the context of Agriculture 4.0?
	What information do you think is needed for a farmer to adopt the PA for irrigation in the context of Agriculture 4.0?
**Additional questions**	Is the planning of agricultural operations an easy process for a farmer? If so, for what reason? If not, what are the difficulties?
	What information is needed for planning agricultural operations?
	Is control of agricultural operations an easy process for a farmer? If so, for what reason? If not, what are the difficulties?
	What information is needed for controlling agricultural operations?

## Appendix B. Questionnaire Used in Case Studies

**Table A2:** Questionnaire used in case studies of açaí palm farms.

**Opening**	**Questions**
Farm characteristics	What is the size of the farm?
	What is the size of the cultivated fields?
	What is the distance between the farmer’s house and the cultivation fields?
	What is the distance between the shed of agricultural machinery and the cultivated fields?
Irrigation system	How do you access the water?
	How do you control the water use?
	What irrigation system do you have on your farm?
	How did you select what irrigation system to use?
**TPB Predictors**	**Questions**
Attitude	Are you considering any changes to the irrigation system? How do you make the decision?
	Have you already heard of the use of sensors and drones? What do you think? Might it be useful? Why yes? Why not?
Perceived behavioral control	What factors would make it easy for you to adopt sensors and drones?
	What factors would make it difficult for you to adopt sensors and drones?
	What information do you think is needed for the adoption of sensors and drones?
**Additional questions**	Is the planning of agricultural operations an easy process for you? If so, for what reason? If not, what are the difficulties?
	What information is needed for planning agricultural operations?
	Is control of agricultural operations an easy process for you? If so, for what reason? If not, what are the difficulties?
	What information is needed for controlling agricultural operations?

## Appendix C. Factors Affecting the Adoption Resulting from Interviews with Experts

**Table A3:** Factors affecting the adoption resulting from interviews with experts.

TPB Predictors	Categories	Expert Quotes	Factors
	Performance measures	“The adoption of Precision Agriculture in the context of Agriculture 4.0 will allow knowing exactly how much water, where, in which way, at what time, in what location. And with another important factor, the cost of energy”. Expert B.	Water use, energy cost
		“For water use to be efficient, the concept of Agriculture 4.0, specifically referring to variable rate management, is fundamental”. Expert L.	Water use
	Access to data	“You have layers of data, soil characteristics, evapotranspiration, rainfall. All this data can be used to calculate how much water is needed”. Expert H.	Evapotranspiration, rainfall, soil characteristics, irrigation water requirement
Attitude	Operations planning and control	“In the agriculture cycle you have the right time to plant. Agriculture is a window”. Expert B.	Harvest time, irrigation window
		“The farmer can increase the management competency, through sensors, to control if something is damaged, broken, stopped, if a problem happened with agricultural machinery and equipment”. Expert I.	Irrigation control
		“I believe that, as agriculture is getting closer to the industrial model, the production planning and control tools would need to be better known by farmers. For example, using production scheduling tools and Gantt chart, controlling when there is a deviation from the planning”. Expert A.	Operations planning, operations control, operations scheduling
		“Regarding the planning of agricultural operations, when we compare production in the industrial and agricultural system, the processes are very similar, excluding the weather issue”. Expert F.	Operations planning, weather
	Changes	“In my opinion, among the essential changes, is the management, quantitative, based on costs”. Expert D.	Management
		“In the area of operations, there will be a management issue: the qualification of the farmer to adopt, understand and use the new technologies”. Expert F.	Management
		“In a large area, where for example a central pivot is used, the farmer can increase the management competency through sensors, which provide data to create a map of that area and to apply a variable rate irrigation”. Expert I.	Management
Perceived behavioral control	Performance measures	“If the farmers know better how Agriculture 4.0 works and are convinced about the advantages of cost, there is no cultural obstacle for adopting”. Expert C.	Costs
	Operations planning and control	“For agricultural operations planning, the information relative to necessary resources, at the level of inputs, hardware, people, is required”. Expert F.	Operations planning, farm resources
		“The mobility degree of machinery and equipment is a challenge that depends on farm size and tools the farmer has”. Expert I.	Mobility degree of production facilities, farm size
		“The coverage of agricultural field is more complicated than in industrial plant, where you have more control. Within a farm it is more difficult and complicated, the infrastructure must be much larger in terms of connectivity”. Expert I.	Coverage of agricultural field, operations control
